# Predictive Biomarkers for Immunotherapy in Endometrial Carcinoma

**DOI:** 10.3390/cancers17152420

**Published:** 2025-07-22

**Authors:** Cristina Pizzimenti, Vincenzo Fiorentino, Ludovica Pepe, Mariausilia Franchina, Chiara Ruggeri, Alfredo Ercoli, Giuliana Ciappina, Massimiliano Berretta, Giovanni Tuccari, Antonio Ieni

**Affiliations:** 1 Section of Pathology, Department of Human Pathology in Adult and Developmental Age ‘Gaetano Barresi’, University of Messina, 98125 Messina, Italy; cristinapizzimenti86@gmail.com (C.P.); vincenzo.fiorentino@unime.it (V.F.); ludopepe97@gmail.com (L.P.); mariausilia.franchina@studenti.unime.it (M.F.); tuccari@unime.it (G.T.); 2 Section of Gynecology and Obstetrics, Department of Human Pathology in Adult and Developmental Age ‘Gaetano Barresi’, University of Messina, 98125 Messina, Italy; chiararug91@gmail.com (C.R.); alfredo.ercoli@unime.it (A.E.); 3 Section of Experimental Medicine, Department of Medical Sciences, University of Ferrara, Via Fossato di Mortara, 70, 44121 Ferrara, Italy; giuliana.ciappina@unife.it; 4 Section of Oncology, Department of Clinical and Experimental Medicine, University of Messina, 98125 Messina, Italy; massimiliano.berretta@unime.it

**Keywords:** endometrial carcinoma, immunotherapy, mismatch repair deficiency, immune checkpoint inhibitor, targeted therapies

## Abstract

Endometrial carcinoma (EC) is the most common gynaecological malignancy in developed nations, exhibiting significant molecular heterogeneity that impacts prognosis and treatment response, particularly in advanced or recurrent settings. Traditional classification is increasingly supplemented by molecular subtyping (*POLE*-ultramutated, MSI-high/dMMR, NSMP, p53-mutated/CNH), which provides crucial prognostic information and predicts benefit from immunotherapy. This review summarizes the landscape of predictive biomarkers for immune checkpoint inhibitor (ICI) therapy in EC, emphasizing a new therapeutic scenario for advanced and recurrent EC. Mismatch repair deficiency (dMMR) or high microsatellite instability (MSI-H), leading to high tumor mutational burden (TMB) and increased neoantigen production, is the most established predictor, resulting in FDA approvals for pembrolizumab and dostarlimab in this subgroup. *POLE* mutations also confer hypermutation and high immunogenicity, predicting a favorable ICI response. Other biomarkers, including PD-L1 expression and TMB, show variable correlation with response and require further standardization. The tumor immune microenvironment, including tumor-infiltrating lymphocytes (TILs), also influences treatment outcomes. Clinical trials have demonstrated significant survival benefits for ICIs combined with chemotherapy (e.g., dostarlimab/pembrolizumab + carboplatin/paclitaxel) in first-line settings, especially for dMMR/MSI-H EC, and for ICI combinations with targeted agents (e.g., lenvatinib + pembrolizumab) in previously treated patients. Integrating molecular classification and validated biomarkers is essential for optimizing patient selection and developing personalized immunotherapy strategies for EC.

## Simple Summary

Endometrial carcinoma represents the most common gynaecological malignancy worldwide, with an unfavourable prognosis in advanced and recurrent stages. Together with traditional morphological classification, a new molecular subtyping approach has been proposed for endometrial carcinoma, thereby influencing therapeutic strategies. The aim of the present review is to analyse predictive biomarkers for an innovative immunotherapy in endometrial carcinomas. Consequently, clinical trials regarding the topic have been reported and discussed in the light of the morphological and molecular neoplastic parameters. Emerging biomarkers, including immune gene expression profiles, intratumoral lymphocytes, and characteristics of the tumor microenvironment, are increasingly being recognized for their potential to refine predictive models. As some open questions are still to be considered by experts on endometrial carcinomas, a criticism regarding the limitation of biomarkers and research gaps needs to be addressed in the future.

## 1. Introduction

Endometrial carcinoma (EC) represents the most prevalent gynecological malignancy in industrialized nations. Globally, it ranks as the sixth most common neoplasm in the female sex and the second most frequently diagnosed tumor affecting the female genital tract, with 420,242 newly diagnosed cases in 2022 [1,2]. In this pathology, several risk factors have been identified, including early menarche, late menopause, nulliparity, obesity, and the use of tamoxifen, all of which contribute to higher oestrogen concentrations that may promote tumorigenesis [2,3]. While the majority of patients are diagnosed with early-stage disease confined to the uterus, exhibiting a 5-year survival rate exceeding 95%, the other ones with advanced or recurrent disease face a significantly poorer prognosis, with a 5-year survival rate below 20% [1,2,3,4,5,6,7,8]. The traditional categorization of ECs in type I (endometrioid) and type II (serous) tumors, based on the Bokhman classification, has been of fundamental importance to risk stratification and guiding clinical decisions for decades [9,10,11].

However, the Cancer Genome Atlas Research Network (TCGA) identified four molecular subgroups of ECs in 2013 based on the molecular features of these tumors: (1) The *POLE* (polymerase epsilon) ultramutated group, distinguished by an exceptionally elevated mutational burden, stemming from pathogenic variants within the *POLE* gene; (2) The microsatellite instability-high (MSI-H) or hypermutated subtype, exhibiting a significant accumulation of mutations, particularly within repetitive DNA sequences known as microsatellites; (3) The copy-number low (CNL), predominantly encompassing low-grade endometrioid carcinomas and characterized by a paucity of somatic copy-number alterations; (4) The copy-number high (CNH), including serous carcinomas and high-grade endometrioid carcinomas, demonstrating a substantial prevalence of genomic copy-number alterations [12]. Subsequently, the ProMisE and TransPORTEC consortium independently developed a pragmatic approach for EC classification using immunohistochemical markers for p53 and mismatch repair deficient (dMMR) and next-generation sequencing (NGS) for detection of pathogenic *POLE* mutations in order to easily reproduce TGCA subtypes [12,13,14]. This development prompted a modification of the 5th edition of the World Health Organization (WHO) Classification of Female Genital Tumours, supporting the integration of established molecular characteristics into the assessment of ECs to provide significant prognostic insights [15].

In 2023, the International Federation of Gynecology and Obstetrics (FIGO) put forward an updated staging system. This new system emphasizes key diagnostic elements, including histological subtype and grade, the presence of lymphovascular space invasion (LVSI), and specific molecular changes [15]. The swift progress in molecular biology techniques has broadened our comprehension of the complex, multi-step nature of EC development, which involves more than just alterations in individual oncogenes and tumor suppressor genes [15].

**Figure 1:**
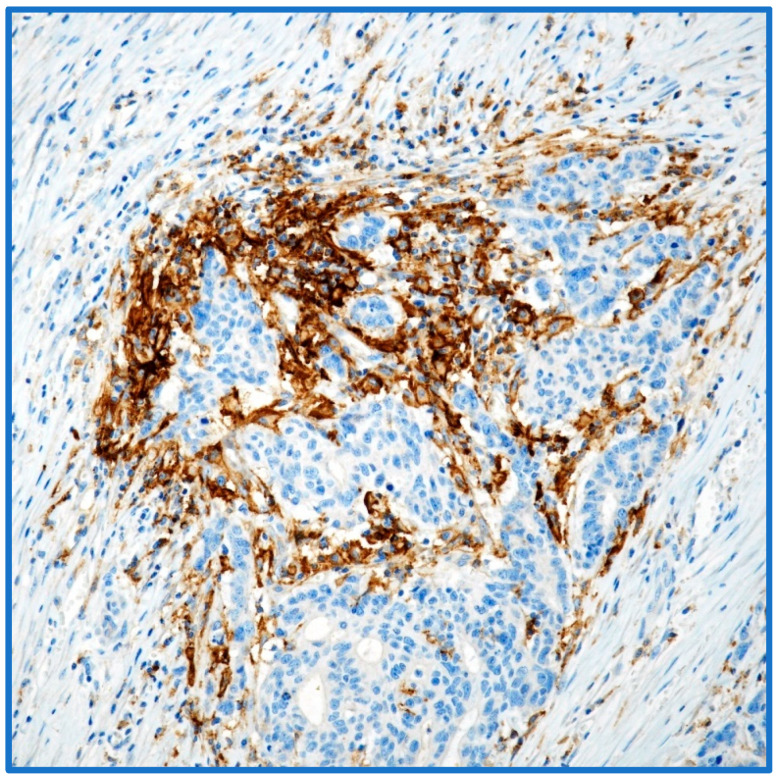
PD-L1 immunoexpression in the endometrioid adenocarcinoma (immunohistochemistry (IHC) and Mayer’s Haemalum counterstain, ×20).

Traditionally, platinum-based chemotherapy has been the mainstay first-line treatment for advanced EC (AEC) [16]. However, recent improvements in understanding the molecular landscape of EC and the role of the immune system in tumor control have moved towards the development of targeted therapies and immunotherapies [5]. Specifically, immune checkpoint inhibitors (ICIs), which target the programmed cell death protein 1 (PD-1) or its ligand (PD-L1), have shown promising results in a subset of EC patients [17]. The rationale behind using ICIs in EC comes from the observation that certain molecular subtypes of EC, particularly those with defects in DNA mismatch repair (dMMR) or microsatellite instability-high (MSI-H), exhibit a high mutational burden, leading to increased neoantigen production and enhanced immunogenicity [18]. These tumors are more likely to be recognized and attacked by the immune system, making them susceptible to ICI therapy [18]. Moreover, the tumor immune microenvironment (TIME) plays a crucial role in modulating the response to immunotherapy [18,19]. Factors such as PD-L1 expression, the presence of tumor-infiltrating lymphocytes (TILs), particularly CD8^+^ T cells, and the overall immune contexture of the tumor are being studied as potential predictive biomarkers [17,19]. PD-L1 expression on tumor cells or immune cells has been associated with improved responses to ICIs in some cancers, but its predictive value in EC is still under investigation (Figure 1).

Likewise, the abundance and spatial arrangement of TILs, particularly CD8^+^ T cells, are correlated with more favourable clinical results in EC patients undergoing immunotherapy. EC should be recognized not as a monolithic disease but rather as a collection of diverse pathologies, each characterized by distinct genomic, molecular, and biological profiles. This concept is underscored by the comprehensive analysis conducted by The Cancer Genome Atlas (TCGA), which has identified several molecular subtypes of EC [12,13,14]. Therefore, for this reason, not all patients respond to immunotherapy, highlighting the need for predictive biomarkers to identify those most likely to benefit and potentially spare those unlikely to respond from unnecessary toxicity and costs [20]. The goal of this article is to provide a comprehensive overview of predictive biomarkers for immunotherapy in EC, including already established markers, emerging candidates, and the challenges associated with their clinical practice.

## 2. Molecular Subtypes of EC

EC is a heterogeneous disease, and its molecular classification has become increasingly important in guiding treatment decisions, including the use of immunotherapy. The molecular subtypes of EC, as defined by TCGA first and the PROMISE consortium later, include *POLE*-ultramutated (*POLE*-mut), microsatellite instability-high (MSI-H)/mismatch repair-deficient (MMRd), copy-number low (also known as “no specific molecular profile” or NSMP), and copy-number high (CNH or p53-mutated) [12,13,14]. Each subtype has distinct molecular features and clinical behaviours, which can influence the response to immunotherapy (Table 1).

### 2.1. POLE-Ultramutated (POLE-mut)

*POLE* mutations are found in 5–8% of all ECs and in about 12.1% of high-grade tumors, including undifferentiated carcinomas and carcinosarcoma [21]. These tumors harbour mutations in the *POLE* gene, which encodes the catalytic subunit of DNA polymerase epsilon [21]. This leads to an ultra-high mutation rate (ultramutation) due to defective DNA proofreading [21]. *POLE*-mut ECs are characterized by high mutation rates, which promote the production of a large number of neoantigens that induce a strong immune response and a favourable response to ICI therapy [23]. NGS is the most comprehensive and widely used approach for analyzing these neoplasms, offering high sensitivity and the ability to detect both known and novel mutations in the *POLE* gene, particularly in the exonuclease domain, where most pathogenic mutations occur [26]. However, studies have shown that *POLE*-mutated ECs have favourable outcomes and may benefit significantly from immunotherapy, although further clinical trials are needed to confirm this [31].

### 2.2. Mismatch Repair Deficiency (dMMR)/Microsatellite Instability-High (MSI-H)

**Figure 2:**
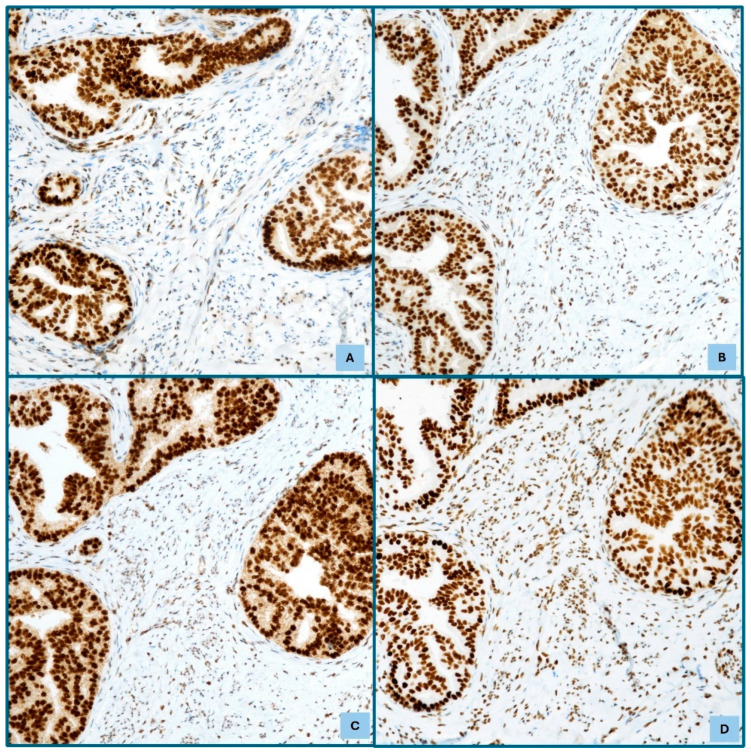
Immunohistochemical expression of MLH1 (**A**), PMS2 (**B**), MSH2 (**C**), and MSH6 (**D**) in MSI stable endometrioid adenocarcinoma (IHC, Mayer’s Haemalum counterstain, ×20).

The dMMR group represents approximately 30% of ECs, and approximately 2–5% of EC cases are attributed to Lynch Syndrome (LS) [22]. This group is distinguished by specific histopathological characteristics. These include an origin in the lower uterine segment and endometrioid differentiation. Furthermore, it is characterized by the presence of severe nuclear atypia, a concurrent undifferentiated component, a high mitotic index, and a significant presence of TILs and/or peri-tumoral lymphocytes (≥40 TILs/10 HPFs). Additional features consist of notable morphological heterogeneity, substantial or focal LVSI, deeper myometrial invasion, and the concurrent presence of ovarian carcinoma, especially of the clear cell or endometrioid subtypes [22]. The dMMR group exhibits microsatellite instability (MSI), a condition resulting from defective DNA mismatch repair, which is crucial for correcting errors that occur during DNA replication [22,24]. This peculiar condition results in high mutation rates, frequent insertions and deletions, and low copy-number variations, particularly in repetitive DNA sequences called microsatellites, resulting in MSI. This hypermutated state generates numerous neoantigens, making the tumor cells more visible to the immune system [24]. MSI arises from either somatic or germline mutations, and the former are responsible for 85% of MSI cases [24]. When germline mutations affect MMR genes, this can lead to disorders such as constitutional mismatch repair deficiency (CMMRD) or LS. LS is an autosomal dominant condition associated with an increased risk of various cancers [24,30] whose definitive diagnosis relies on the detection of pathogenic variants in MMR genes through germline sequencing, but IHC is the first step for screening in EC (Figure 2).

**Figure 3:**
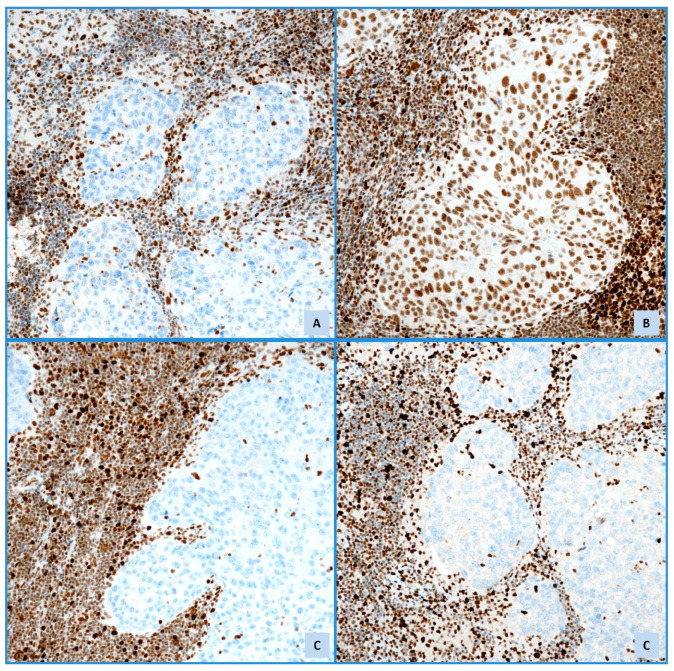
Immunohistochemical expression of MLH1 (**A**), MSH2 (**B**), MSH6 (**C**), and PMS2 (**D**) in MSI unstable lymph node metastatic high-grade endometrioid adenocarcinoma (IHC, Mayer’s Haemalum counterstain, ×20).

Universal tumor screening is increasingly being adopted to identify individuals at risk of LS. EC is often associated with the dMMR/MSI-H phenotype, and the use of IHC is recommended for assessing MMR status (Figure 2 and Figure 3). IHC is widely available and inexpensive, with MMR IHC for MLH1, PMS2, MSH2, and MSH6 being the gold standard surrogate testing method [30]. Identifying the loss-of-expression pattern of ECs with dMMR/MSI-H status provides information on the altered gene, and results in three benefits: detecting patients at higher risk of presenting LS, providing prognostic information as a surrogate marker for dMMR EC, and holding predictive value [30] (Figure 3). Universal testing for dMMR/MSI-H is now recommended for all endometrial tumors [32].

According to Addante et al. [22], dMMR ECs are thought to be the best candidates for immunotherapy because of their rich immune infiltration and high mutation burden. Every dMMR tumor has high microsatellite instability (MSI-H), a high mutational load (H-TMB: 10–100 mutations per megabase), and a rapid accumulation of genomic mutations [32]. These tumors are extremely immunogenic, exhibiting high levels of lymphocyte infiltration and robust expression of immunological checkpoints, and they include hundreds to thousands of mutations [25,32]. In dMMR tumors, programmed death ligand 1 (PD-L1) can be present on the tumor cell surface. Concurrently, lymphocytes that infiltrate the tumor often show increased levels of immune checkpoint proteins, including lymphocyte-activation gene 3 (LAG-3), cytotoxic T-lymphocyte-associated protein 4 (CTLA-4), and programmed death 1 (PD-1). The high frequency of mutations in dMMR malignancies, particularly frameshift mutations that result in mutant protein neoantigens, may cause immune cell infiltration [22,25].

### 2.3. Copy-Number Low (NSMP)

NSMP ECs are characterized by a low frequency of copy-number alterations and are often associated with PTEN mutations and PIK3CA mutations [27]. They are typically estrogen receptor (ER)-positive and have a relatively stable genome. These tumors account for half of all ECs and have an intermediate prognosis [27,28]. Having a lower TMB and immunogenic potential, NSMP ECs are less likely to respond to immunotherapy [27,28]. However, some studies have suggested that ER-negative NSMP tumors may have a better response to ICIs, although this requires further validation [27,28].

### 2.4. CNH/p53-Mutated

CNH/p53-mutated ECs are characterized by high copy-number alterations and frequent TP53, PPP2R1A, and FBXW7 mutations [29]. They account for 15% of all ECs, but they have a more aggressive clinical course and are associated with 50–70% of mortality [29]. CNH/p53-mutated ECs are often associated with serous or serous-like histology, but can occur in all histological types [29]. Typically, these tumors have a low TMB and are less immunogenic compared to *POLE*-mut and dMMR/MSI-H tumors [29]. As a result, they are less likely to respond to immunotherapy. However, some studies have explored the role of combining ICIs with other therapies, such as PARP inhibitors, to enhance the immune response in these tumors [12].

## 3. Tumor Mutational Burden

TMB is a measure of the total number of somatic mutations per megabase (mut/Mb) of DNA in a tumor obtained through Next Generation Sequencing (NGS) [33]. TMB-high (TMB-H) is the term used to describe cancers having more than 10 mutations/Mb [33]. A higher TMB is generally associated with increased neoantigen production and enhanced immune recognition [34]. Specific cancer subtypes, such as *POLE*-mutant and MMRd ECs, are typically TMB-H neoplasms characterized by immunogenic tumor microenvironments [25]. TMB is under investigation as a potential predictive biomarker across various cancer types, including EC [35]. Generally, a correlation is observed between TMB levels and response to anti-PD-1 therapies [36]. A post-hoc analysis of the GARNET trial showed that patients with TMB-H EC experienced high responses to dostarlimab, irrespective of MMR or MSI status [37].

Conversely, the optimal cutoff value for defining TMB-H in EC remains to be standardized, and different tumor types may require different cutoffs [38]. The correlation between TMB and ICI response is not clear. High TMB is linked to better survival, higher tumor grades, and certain EC pathological types, and TMB-related genes may be potential therapeutic targets for EC [38]. Patients with high TMB had more tumor-infiltrating lymphocytes, suggesting TMB could predict response to immunotherapy [38]. It has been concluded that targeting these genes with drugs could be a promising new approach to EC treatment [38].

On the other hand, McGrail et al. [39] examined the relationship between TMB and the effectiveness of immune checkpoint blockade (ICB) therapy; their research indicated that in specific cancer types, including melanoma, lung, and bladder cancer, a positive correlation was observed between CD8 T-cell levels and neoantigen load [39]. Furthermore, TMB-H tumors demonstrated a notably greater response rate to ICB [39]. Conversely, in cancer types where no such relationship between CD8 T-cell levels and neoantigen load was evident, such as breast cancer, prostate cancer, and glioma, TMB-H tumors did not exhibit an improved response rate to ICB [39]. Hence, it may be suggested that TMB-H may not be a reliable biomarker for ICB response across all cancer types [39]. Further research is needed to determine the optimal TMB threshold for different cancer types in order to verify the existence of a potential relationship between TMB, immune infiltrates, and prognosis in EC [40].

## 4. Immune Micro-Environment in EC

The immune system plays a crucial role in the normal endometrium, particularly within endometrial epithelial cells [41]. It acts as a physical barrier and produces defensins, immune mediators and antigens [41,42]. Both the innate and adaptive immune systems contribute to pathogen elimination through the production of inflammatory cytokines, modulating immune reactions [41,42]. These immunological activities are influenced by sex hormones, notably estradiol and progesterone, which show variable levels throughout the menstrual cycle. The immune system encompasses a range of elements that sustain normal physiological functions. It also moderates immune activity to guard against maternal rejection of the fetus and shields the vulnerable endometrium from possible infections during menstruation [41,42]. PD-1, a protein that inhibits autoimmunity, limits infection-related damage to healthy tissues, and fosters self-tolerance, can affect the capacity of T cells to combat cancer and infectious agents [41,42,43].

Regarding EC, TIM plays a dual role both in suppressing and promoting tumor growth, which is crucial to understand and develop effective immunotherapies [41,42,43]. It is well known that immune cells identify and eliminate cancer cells, contributing to immunosurveillance [17,19]. By contrast, TIME can promote tumor progression and immune evasion [17,19]. The most critical TIME components are TILs, particularly CD8^+^ cytotoxic T cells, which are a key component of the antitumor immune response, recognizing and eliminating cancer cells [17,19]; in the meantime, CD4^+^ T cells organize the immune response through the activation of other immune cells and cytokine production [17,19]. Among TIME components, regulatory T cells (Tregs) suppress immune response and promote immune evasion, inhibiting CD8^+^ T cells and other effector immune cells, creating an immunosuppressive TIME [17,19]. Otherwise, tumor-associated macrophages (TAMs) can have both pro-tumor and antitumor effects, depending on their polarization, with M1 macrophages antitumor and promoters of inflammation, while M2 macrophages pro-tumor and promoters of angiogenesis, tissue remodeling and immune suppression [17,19].

Finally, myeloid-derived suppressor cells (MDSCs) are another immunosuppressive cell population that inhibits T cell function and promotes tumor progression [17,19]. All interactions between EC cells and TIME often involve immune checkpoint pathways, such as the PD-1/PD-L1 axis [17,19]. Specifically, the upregulation of PD-L1 expression on EC cells and their binding with PD-1 on T cells leads to the inhibition of the cytotoxic capabilities of T cells, their exhaustion and the reduction of cytokine production with antitumor abilities. Moreover, tumor cells can produce cytokines such as TGF-β, IL-10 or IL-6, that can contribute to immune suppression and tumor progression through the inhibition of T cell activity and the promotion of the differentiation of Tregs [17,19].

## 5. PD-L1 Expression

PD-1, a 288-amino acid transmembrane protein, is expressed on diverse immune cell types and belongs to the CD28 superfamily; its synthesis is directed by the programmed cell death protein 1 (PDCD1) gene. These cell types include activated T lymphocytes, natural killer cells, B cells, macrophages, dendritic cells (DCs), and monocytes [41]. Functionally, PD-1 downregulates both adaptive and innate immune pathways. Its expression is governed by transcription factors including NFAT, FOXO1, and IRF9 [44,45]. Furthermore, the upstream regulatory sequences B and C, also referred to as CR-B and COR-C, are critical for modulating PD-1 gene activity [44,45,46]. CR-C has a binding site associated with NFATc1 in TCD4 and TCD8 cells, while c-FOS interacts with specific sites within the CR-B region, leading to an increase in PD-1 production following antigen recognition and subsequent activation of naïve T-cell receptors.

PD-1 has both beneficial and detrimental effects [44,45,46]. On the positive side, it curbs harmful immune responses and maintains immunological tolerance [44,45,46]. However, activation of PD-1 can hamper the protective immune response, leading to the development of malignant cells [44,45,46]. The PD-1 receptor can bind to a number of ligands, including PD-L1, a transmembrane protein expressed on tumor cells and immune cells [47]. This protein interacts with T cell PD-1, triggering a co-inhibitory signal in T cells as tumor cells increase the synthesis of PD-L1 [47]. This process promotes the growth of tumors by allowing neoplastic cells to avoid destruction by T-cell cytolysis. For immune checkpoint inhibitors (ICIs), the pathway is the main focus.

Specific inhibitors targeting either PD-1 or PD-L1 can block the interaction between the PD-L1 ligand and the PD-1 receptor [44]. PD-L1 is a protein commonly expressed on the surface of macrophages, activated T cells, B cells, DCs, and certain epithelial cells, particularly when inflammatory signals are present. Tumor cells leverage PD-L1 expression to avoid antitumor immune surveillance; this is recognized as an adaptive immune evasion mechanism [44]. It is linked to an immune microenvironment characterized by CD8^+^ T cells, Th1-type cytokines, various chemical factors, interferon synthesis, and unique gene expression profiles [48]. In ovarian cancer cells, interferon-gamma (IFN-γ) promotes an increase in PD-L1 expression, which aids disease progression [48,49].

However, inhibiting the IFN-γ receptor 1 has been shown to decrease PD-L1 concentrations in mouse models of acute myeloid leukaemia [48,49]. IFN-γ also upregulates protein kinase D isoform 2 (PKD2), an enzyme that significantly influences PD-L1 regulation [48,49]. NK cells generate IFN-γ by activating the Janus kinase (JAK)1, JAK2, and signal transducer and activator of transcription (STAT)1 pathway; this process elevates PD-L1 expression on the surface of tumour cells [49,50]. Furthermore, studies using melanoma cells have demonstrated that IFN-γ secreted by T cells can modify PD-L1 expression [49,50,51]. IFN-γ, released by both T lymphocytes and NK cells, stimulates the upregulation of PD-L1 on the surface of target cells, encompassing tumor cells [48,49,50,52].

### PD-L1 Expression in EC

The role of PD-L1 expression as a predictive biomarker for response to ICIs has been studied [46,51,53] though its utility in EC remains a topic of discussion [39]. Current research indicates that first-line treatment with anti-PD-1/PD-L1 agents results in response rates from 20% to 65% in tumors positive for PD-L1 across various cancer types, including EC [52]. Conversely, tumors that lack PD-L1 expression show response rates between 0% and 17% in diverse tumor types [52]. Expression of PD-L1 within the tumor microenvironment is considered an important biomarker for identifying potential immunotherapy responders [52,54]. In their study of 132 patients with microsatellite stable (MSS) grade 2 endometrioid EC, Crumley et al. found that PD-L1 was positive in 48% (63/132) of the tumors.

Furthermore, 16% (21/132) of the total cases were identified as a ‘PD-L1 high’ subset, which was characterized by more diffuse and/or especially strong PD-L1 expression and was associated with significantly higher numbers of tumor-associated CD3^+^ and CD8^+^ lymphocytes [55]. This subgroup of EC shares similarities with dMMR/MSI EC, a class of EC with microsatellite genetic instability [55], demonstrating increased levels of tumor-associated CD3^+^ and CD8^+^ lymphocytes, a trait also observed in dMMR/MSI EC [55]. In 2019, the European Society for Medical Oncology (ESMO) conducted an extensive study assessing the link between MSI status and PD-L1 expression across various malignancies, including EC. This research provided an understanding of how these factors interact in different cancers and may potentially shape treatment decisions [23]. The conclusions from both the original research on the grade 2 endometrioid carcinoma subgroup and the more comprehensive ESMO investigation allow a better understanding of the multifaceted relationship involving PD-L1 expression, MSI, and immune cell infiltration within various tumor types [23].

Microsatellite instability (MSI) and positive PD-L1 status are common in endometrioid cancer (EC), with only 3.1% of patients exhibiting these characteristics [23]. Research by Vanderwalde et al. [56] showed that similar percentages of PD-L1 positivity were found in the entire study group and in MSI-H patients (25.4% and 26%, respectively). Multiple investigations [57] have also linked PD-L1 expression to a greater occurrence of high-grade and non-endometrioid EC. Furthermore, other studies [23] have reported higher PD-L1 expression in *POLE* and dMMR subgroups compared to NSMP and p53 mutation subgroups. PD-L1 expression within immune cells has been correlated with factors including deep myometrial invasion, LVSI, and the non-endometrioid histological subtype of EC [58]. In addition, high-grade tumors have a higher expression of PD-L1 in tumor cells compared to low-grade tumors [58].

The relationship between PD-L1 expression and EC prognosis has been a topic of interest, with inconsistent findings regarding tumor cells and immune cells’ associations with survival. Recent investigations have underscored the complex role of PD-L1 in this context. For example, Zong et al. [58] demonstrated that PD-L1 expression on tumor cells was linked to a more favourable prognosis for patients with FIGO stages II–IV non-endometrioid EC, a correlation not observed for PD-L1 on immune cells [58]. Separately, Zhang et al. [59] identified high PD-L1 expression on tumor cells as an independent factor for predicting better overall survival (OS), whereas increased PD-L1 levels on immune cells correlated with worse OS [59]. Furthermore, Yamashita et al. [60] also connected PD-L1 expression on tumor cells with longer progression-free survival (PFS) [60]. In contrast, Chew et al. [61] reported conflicting data, indicating that tumor cells’ PD-L1 expression was significantly tied to poor survival [61]. Finally, Kucukgoz Gulec et al. [62] found that in non-endometrioid EC, PD-L1 expression on tumor cells was associated with shorter survival durations [62].

## 6. Immunotherapy for EC

To date, combined chemotherapy with carboplatin plus paclitaxel is the chosen first-line treatment for AEC, with an overall response rate of 50–60% and a median PFS of 1 year [63], followed by a second-line treatment based on single drug chemotherapy. Since the advent of EC molecular classification, the relevance of immunotherapy has increased, especially for those highly immunogenic subtypes such as dMMR/MSI ECs [64,65]. In particular, pembrolizumab, an anti-PD-1 antibody, was the first site-agnostic cancer drug approved by the FDA for the treatment of MSI-H or dMMR solid tumors, including EC [64]. Dostarlimab, another anti-PD-1 antibody, is also approved for dMMR/MSI-H EC, both as a single agent and in combination with chemotherapy [66].

### 6.1. Clinical Immunotherapeutic Trials in EC

Several clinical trials have demonstrated the efficacy of ICIs in dMMR/MSI-H EC [24] (Table 2). The multicohort Ib KEYNOTE-028 (NCT02054806) study showed an ORR of 13% with pembrolizumab in PD-L1-positive AEC, demonstrating a favourable safety profile and durable antitumor activity in a subgroup of patients with heavily pretreated advanced PD-L1-positive EC [54,66,67]. The KEYNOTE-158 study showed that pembrolizumab had antitumor effects in patients with dMMR/MSI-H [68]. The GARNET study demonstrated significant activity of dostarlimab in patients with dMMR recurrent or AEC [36]. The DUO-E trial (GOG-3041/ENGOT-EN10), consisting of carboplatin/paclitaxel plus durvalumab, followed by maintenance durvalumab with or without Olaparib, showed survival benefits in terms of a PFS advantage, with a positive clinical impact, combining immunotherapy with chemotherapy in dMMR EC [69]. Hasegawa et al. [70] investigated the effectiveness and tolerability of nivolumab, a human monoclonal antibody targeting PD-1, in individuals with advanced or recurrent uterine cervical cancer, EC, or soft tissue sarcoma (STS). The nivolumab dose was 240 mg every two weeks. The main outcomes measured were the objective response rate (ORR), OS, PFS, and safety [70]. Biomarkers such as PD-L1 expression and MSI status were also evaluated for their predictive value [70]. ORRs were 25% for cervical cancer, 23% for EC, and 0% for STS [70]. The median PFS was 5.6, 3.4, and 1.4 months, respectively, while 6-month OS rates were 84% (cervical cancer), 73% (EC), and 86% (STS) [70]. Patients with PD-L1-positive tumors showed a superior ORR when compared to individuals whose tumors were PD-L1-negative [70]. Moreover, nivolumab presented a tolerable safety profile in all evaluated groups and demonstrated therapeutic effectiveness in cases of uterine cervical cancer or EC [70]. Konstantinopoulos et al. [71] assessed the impact of ICB with pembrolizumab in EC characterized by either proficient MMR (pMMR) or dMMR. The study included 33 patients; the dMMR cohort had no *POLE*-mutated tumors, and all pMMR tumors were non-*POLE*-mutated [71]. The primary endpoints were objective response (OR) and PFS at 6 months (PFS6) [71]. Avelumab was dosed to patients via intravenous infusion on a bi-weekly schedule [71]. This regimen was maintained until there was evidence of disease advancement or the development of intolerable adverse events [71]. The pMMR arm was discontinued due to lack of efficacy, whereas the dMMR arm achieved its primary endpoint of four ORs with only 17 patients enrolled [71]. Among 15 patients who started avelumab, four achieved an OR and six had PFS6, with four of these responses ongoing. Notably, responses occurred even without PD-L1 expression [71]. The study concluded that avelumab showed promising efficacy in dMMR EC irrespective of PD-L1 status, highlighting IHC for MMR assessment as a valuable patient selection tool [71].

Conversely, avelumab’s activity was limited in pMMR/non-*POLE*-mutated ECs. Antill et al. [72] explored the activity of durvalumab, an anti-PD-L1 antibody, in women with AEC, distinguishing between pMMR and dMMR tumors. The study population comprised pMMR patients who had progressed after one to three chemotherapy lines, and dMMR patients who had progressed after zero to three chemotherapy lines [72]. The primary outcome was OR based on Response Evaluation Criteria in Solid Tumors (RECIST) V.1.1, modified for immune-based therapies [72]. Seventy-one women participated, with a median follow-up of 19 months (longer in dMMR than pMMR) and a median age of 67 years [72]. The objective tumor response rate (OTRR) in the dMMR group was 47%; 58% of these women received it as first-line therapy, and 39% as second-line [72]. Median PFS was 8.3 months in the dMMR cohort, and the 12-month overall survival rate was 71% for dMMR versus 51% for pMMR [72]. Immune-related adverse events, mostly grades 1–2, affected 14 women. The authors concluded that durvalumab monotherapy demonstrated encouraging activity and acceptable safety in dMMR AEC, but its efficacy was restricted in pMMR cases [72]. Another study evaluated the antitumor effects and safety of dostarlimab, an investigational anti-PD-1 antibody, in patients with dMMR EC [73]. This study involved 104 women with this type of cancer. Among this group, 71 participants had quantifiable disease at the initial assessment and were monitored for a minimum of 6 months [73]. The primary endpoints were ORR and duration of response (DOR), assessed by blinded independent central review using RECIST version 1.1. Results indicated that dostarlimab provided clinically significant and sustained antitumor activity with a manageable safety profile for patients with deficient mismatch repair ECs previously treated with platinum-based chemotherapy [73]. The median DOR was not reached; the estimated probability of maintaining a response was 96.4% at 6 months and 76.8% at 12 months [73]. The most common therapy-associated adverse effects, reaching grade 3 or higher severity, were anemia (2.9%, 3/104), colitis (1.9%, 2/104), and diarrhea (1.9%, 2/104) [73]. The potential for immunotherapy to improve outcomes for individuals with dMMR EC when combined with standard adjuvant therapies is still an open question, currently under investigation in the RAINBO clinical trial program.

This program aims to assess four molecular class-directed adjuvant treatment strategies after surgical resection for women with EC [74]. The hypothesis is that these strategies will lead to better clinical outcomes and lessen the toxicity from unnecessary treatments [74]. The broader RAINBO research initiative will enhance understanding of predictive and prognostic biomarkers, thereby improving prognostication and treatment decisions. The RAINBO program encompasses four international clinical trials: p53abn-RED for women with invasive stage I-III p53-mutated EC, MMRd-GREEN for stage II or III dMMR EC, NSMP-ORANGE for estrogen receptor-positive stage II or III NSMP EC, and *POLE*-mut-BLUE for stage I-III *POLE*-mut EC [74]. Primary endpoints include 3-year recurrence-free survival (RFS) for the p53abn-RED, MMRd-GREEN, and NSMP-ORANGE trials, and 3-year pelvic recurrence for the *POLE*-mut-BLUE trial [74]. The trial plans to enroll 554 patients, with the entire program targeting a total sample size of approximately 1600 cases. The goal is to advance knowledge of biomarkers to refine prognostication and treatment allocation for EC patients [74]. A phase Ib/II study has published its final primary efficacy analysis for a cohort of AEC patients treated with lenvatinib plus pembrolizumab [75,76]. Patients received oral lenvatinib 20 mg daily and intravenous pembrolizumab 200 mg every 3 weeks, in 3-week cycles [75,76]. The ORR at 24 weeks (ORRWk24) was designated as the primary endpoint, with DOR, PFS, and OS serving as secondary endpoints. Tumor responses were assessed using immune-related RECIST criteria. At the time of data cutoff, 108 patients with previously treated EC were enrolled, with a median follow-up of 18.7 months [75,76]. The ORRWk24 was 38.0% overall: 63.6% in patients with MSI-H tumors and 36.2% in those with MSS tumors. Median DOR, PFS and OS were 21.2 months, 7.4 months, and 16.7 months, respectively.

Regarding safety, 66.9% (83/124) of patients experienced Grade 3 or 4 adverse events considered attributable to the treatment [76]. Subsequently, the same research group presented the final analysis of OS, PFS, ORR, and safety from the open-label, randomized phase III Study 309/KEYNOTE-775. This trial involved 827 patients with advanced, recurrent, or metastatic EC, randomly assigned to either oral lenvatinib 20 mg daily plus intravenous pembrolizumab 200 mg every 3 weeks, or a physician’s choice of chemotherapy. The study demonstrated that lenvatinib plus pembrolizumab offered superior OS, PFS, and ORR compared to chemotherapy. Furthermore, no new safety concerns emerged, and the combination continued to show enhanced efficacy and a manageable safety profile in patients with previously treated AEC [74,75,76]. The findings highlight the importance of considering the specific subgroups when evaluating the effectiveness of lenvatinib plus pembrolizumab [74,75,76].

In a phase II clinical trial [77], Lheureux and colleagues evaluated a therapeutic strategy for women with recurrent EC. Participants were assigned randomly in a 2:1 ratio to receive either nivolumab (a checkpoint inhibitor) plus cabozantinib (an antiangiogenic agent) in Arm A (n = 36) or nivolumab as a monotherapy in Arm B (n = 18). A distinct group, Arm C, administered the combination treatment and comprised individuals with carcinosarcoma or those who had previously undergone ICI therapy. The primary endpoint was PFS, assessed using RECIST criteria. The median PFS was observed to be 5.3 months in Arm A, compared to 1.9 months in Arm B. This result met the predefined statistical significance thresholds. The most frequently reported treatment-associated adverse effects in Arm A included diarrhea (50%), increased aspartate aminotransferase (47%), and elevated alanine aminotransferase (42%).

A comprehensive baseline cytometry by time of flight (CyTOF) analysis was conducted on initial biopsy specimens and sequential peripheral blood mononuclear cell (PBMC) samples from 40 participants across the treatment arms. This analysis identified 35 distinct immune-cell populations. Notably, among individuals in Arm C who had prior immunotherapy, those who did not experience disease progression exhibited significantly greater proportions of activated tissue-resident (CD103^+^CD69^+^) ɣδ T cells relative to those whose disease progressed. The study concluded that combining cabozantinib with nivolumab resulted in substantial improvements in clinical outcomes for heavily pretreated EC. Considering these results, further research is required to identify a specific subgroup of immunotherapy-pretreated EC patients, possibly distinguished by their baseline immunological characteristics, who could potentially benefit from the integration of antiangiogenic agents. In a separate investigation, Eskander et al. [78] conducted a study with 816 participants who had advanced or recurrent EC. Their results indicated that incorporating pembrolizumab into standard chemotherapy regimens led to a significantly prolonged PFS when contrasted with chemotherapy administered alone [78]. This research was a double-blind, placebo-controlled, randomized, phase 3 trial. In this trial, patients were allocated to receive either pembrolizumab or a placebo, concurrently with combination therapy consisting of paclitaxel plus carboplatin [78]. The patient population was stratified into two distinct cohorts depending on their dMMR or pMMR disease status. The principal outcome measure was PFS. The 12-month analysis demonstrated a 70% difference in PFS favoring the pembrolizumab arm over the placebo arm. Concurrently, the pMMR cohort experienced a median PFS of 13.1 months when treated with pembrolizumab, as opposed to 8.7 months for those receiving the placebo [78]. The observed adverse events were consistent with the known profiles of pembrolizumab and the combination chemotherapy. The authors concluded that the addition of pembrolizumab to conventional chemotherapy provides a more efficacious treatment approach for advanced or recurrent EC [78].

Another study investigating the combination of chemotherapy and immunotherapy in EC was a phase 3, global, double-blind, randomized, placebo-controlled trial conducted by Mirza and colleagues [79]. This study aimed to determine the synergistic efficacy of an ICI (dostarlimab) combined with carboplatin-paclitaxel for EC treatment. Patients meeting eligibility criteria, who presented with primary advanced stage III or IV, or first-recurrence EC, were randomly allocated. They received either dostarlimab (500 mg) or a placebo, in conjunction with carboplatin and paclitaxel administered every three weeks. After completion of the aforementioned regimen, patients proceeded to a treatment phase involving either dostarlimab (1000 mg) or placebo, administered at intervals of six weeks for a period extending up to three years [79]. The primary endpoints were PFS and OS. The findings indicated that within the dMMR-MSI-H subgroup, the cohort receiving dostarlimab demonstrated an estimated 24-month PFS rate of 61.4%. In contrast, the placebo cohort showed a corresponding rate of 15.7% [79]. When considering the entire study population, the 24-month PFS for the dostarlimab arm was 36.1%.

Furthermore, OS rates were 71.3% for those treated with dostarlimab compared to 56.0% for those in the placebo arm [79]. The most frequently reported adverse effects during the treatment period were nausea, alopecia, and fatigue. A higher frequency of severe and serious adverse events was noted in the group receiving dostarlimab. To conclude, the incorporation of dostarlimab with carboplatin-paclitaxel resulted in a significant enhancement of PFS for patients with primary advanced or recurrent EC, showing a particularly pronounced advantage in the dMMR-MSI-H population.

**Table 2:** Summary of Selected Immunotherapy Trials and Agents in EC.

Agent(s)/Trial	Target/Mechanism	Patient Population/Setting	Key Outcomes/Findings	Reference(s)
Pembrolizumab (KEYNOTE-028, -158)	Anti-PD-1	PD-L1 + AEC (028); dMMR/MSI-H solid tumors incl. EC (158).	Showed durable activity and safety in PD-L1 + EC (ORR 13% in 028); Effective in dMMR/MSI-H EC; Led to site-agnostic approval for MSI-H/dMMR tumors.	[24,66,67]
Dostarlimab (GARNET)	Anti-PD-1	Recurrent or advanced dMMR/MSI-H EC.	Clinically meaningful and durable activity with acceptable safety; Approved for dMMR/MSI-H EC.	[36]
Durvalumab + Carboplatin/Paclitaxel, followed by maintenance Durvalumab with or without Olaparib DUO-E trial (GOG-3041/ENGOT-EN10	Anti-PD-1 +	Advanced or recurrent EC (dMMR and pMMR cohorts).	PFS benefit in dMMR, 0.42 [95% CI, 0.22 to 0.80]; 0.41 [95% CI, 0.21 to 0.75]) and pMMR subgroups, 0.77 [95% CI, 0.60 to 0.97]; 0.57; [95% CI, 0.44 to 0.73]); and in PD-L1–positive subgroups, 0.63 [95% CI, 0.48 to 0.83]; 0.42 [95% CI, 0.31 to 0.57].	[69]
Avelumab (Konstantinopoulos et al.).	Anti-PD-L1	Recurrent/persistent dMMR and pMMR EC.	Promising activity in dMMR EC regardless of PD-L1 status. Low activity in pMMR/non-*POLE*-mut EC.	[71]
Durvalumab (Antill et al.)	Anti-PD-L1	AEC: dMMR (0–3 prior lines), pMMR (1–3 prior lines).	Promising activity and acceptable safety in dMMR AEC (ORR 47%). Limited activity in pMMR AEC.	[72]
RAINBO Program	Various (based on subtype)	Adjuvant setting post-surgery for specific molecular subtypes (p53abn, MMRd, NSMP, *POLE*-mut).	Ongoing program investigating molecularly-directed adjuvant therapies to improve outcomes and reduce toxicity.	[74]
Cabozantinib + Nivolumab (Lheureux et al.)	Multi-kinase inhibitor + Anti-PD-1	Recurrent EC (immunotherapy-naïve and prior ICI).	Combination significantly improved outcomes (PFS) in heavily pretreated EC vs. historical controls.	[77]
Pembrolizumab + Chemotherapy (Eskander et al./KEYNOTE-868/NRG-GY018)	Anti-PD-1 + Carboplatin/Paclitaxel	Advanced or recurrent EC (dMMR and pMMR cohorts); first-line.	Significantly longer PFS vs. chemo alone, especially in dMMR cohort (70% reduction in progression/death risk). Benefit also seen in pMMR cohort (median PFS 13.1 vs. 8.7 mo).	[78]
Dostarlimab + Chemotherapy (Mirza et al./RUBY/ENGOT-EN6/GOG3031/NSGO)	Anti-PD-1 + Carboplatin/Paclitaxel	Primary advanced stage III/IV or first recurrent EC (dMMR/MSI-H and overall populations)	Significantly improved PFS vs. chemo alone in both dMMR/MSI-H population (est. 24-mo PFS 61.4% vs. 15.7%) and overall population (est. 24-mo PFS 36.1% vs. 18.1%). Substantial benefit in dMMR/MSI-H.	[79]

EC—Endometrial Carcinoma; AEC—Advanced EC; PD-1—Programmed cell death protein 1; PD-L1—Programmed death-ligand 1; dMMR—deficient Mismatch Repair; pMMR—proficient Mismatch Repair; MSI-H—Microsatellite Instability-High; ORR—Objective Response Rate; PFS—Progression-Free Survival; OS—Overall Survival; ICI—Immune Checkpoint Inhibitor; Chemo—Chemotherapy.

### 6.2. Role of PD-1/PD-L1 Expression in EC

In recent years, research on PD-1/PD-L1 expression in EC has made considerable progress, revealing its role in the tumor microenvironment. However, there are still elusive aspects, indicating gaps in our understanding. One challenge is the inconsistent and inconclusive evidence, with some studies reporting significant expression levels, while others find lower levels due to variations in patient populations, tumor heterogeneity, and differences in laboratory techniques used to assess PD-1/PD-L1 expression. One aspect that is going to be investigated is the lack of PD-1/PD-L1 expression in certain subgroups of EC patients, which is a critical mechanism for immune evasion by tumor cells with deep genetic mutations, epigenetic alterations, or microenvironmental factors. It could be useful for a better understanding of this phenomenon. The complex interaction between PD-1/PD-L1 expression and other immune microenvironment components, such as immune cells, cytokines, and other immuno-modulating factors, further complicates the picture. A thorough comprehension of these immune-cancer interactions is essential for the development of targeted therapies capable of effectively counteracting immune evasion strategies employed by tumors. Treatment with PD-1/PD-L1 inhibitors (ICIs) represents a fundamental component of cancer immunotherapy, significantly altering the landscape of cancer care. The efficacy of ICIs is attributed to their ability to modulate immunity at the tumor site by blocking the PD-1/PD-L1 pathway, thereby enabling immune cells to better eliminate cancer cells within the tumor microenvironment [47,51,53].

Furthermore, ICIs address tumor-induced immune defects by overcoming the immunosuppressive environment that tumors create, allowing the immune system to recognize and destroy malignant cells. They also repair ongoing tumor immunity, restoring and strengthening existing antitumor responses, which makes them effective even in advanced stages like AEC [47]. Despite these successes, the optimal application of ICIs in EC remains uncertain. While high PD-1/PD-L1 expression is often linked to better outcomes, tumors can develop resistance by increasing this expression [47]. Consequently, initial assessment and continuous monitoring of PD-L1 levels during treatment are crucial. However, as the complexities of ICIs in EC remain unelucidated, clinical trials are of paramount importance to fully explore their therapeutic potential. Consequently, continued investigation into immunotherapy and associated genetic profiles in EC is imperative to facilitate the development of personalized targeted therapies and novel treatment strategies. Additional information focusing on patient stratification pointed out IFN-γ signatures as well as cytolytic scores.

In detail, high stimulation of IFN-γ may determine CD8^+^ T-cells apoptosis or induction of PD-L1 expression to prevent cytotoxic T lymphocytes (CTL) migration to the tumor microenvironment (TME), thus lowering immune response [80]. By contrast, low-dose IFN-γ can produce tumor stemness, increasing the risk of tumor metastasis during immunotherapy [81]. Nevertheless, to date, precise and more appropriate concentration of IFN-γ in neoplastic conditions has not yet been determined [80]. Furthermore, it has been reported that the immune cytolytic activity (CYT) index was significantly correlated with the development and response to immunotherapy in EC [81]. Higher CYT was significantly associated with better clinical outcome, more antitumor infiltrating immune cells, fewer somatic copy number alterations, but a higher TMB (Tumor mutational burden) status has been documented [81]. Consequently, CYT-high EC reflected high expression of immune checkpoint (IC) molecules, showing an effective response to IC treatment [81].

## 7. Conclusions and Future Perspectives

The exploration of predictive biomarkers for immunotherapy in EC has made significant strides in recent years, offering promising avenues for improving patient outcomes. Molecular markers such as mismatch repair deficiency, microsatellite instability, and TMB have emerged as critical predictors of response to immunotherapy. These biomarkers have been shown to correlate with improved survival rates in patients treated with ICIs. However, despite these advances, not all patients with dMMR or MSI-high tumors respond uniformly, which underscores the complexity of tumor immunology and the need for a more nuanced approach to biomarker-based patient selection. Emerging biomarkers, including immune gene expression profiles, TILs, and characteristics of the tumor microenvironment, are increasingly being recognized for their potential to refine predictive models. These factors may influence the efficacy of immunotherapies beyond what is currently understood from traditional biomarkers alone. Additionally, the advent of multi-omic technologies, which combine genomic, transcriptomic, and proteomic data, offers the possibility of identifying novel biomarkers that could provide a more comprehensive and personalized strategy for immunotherapy in EC. The ultimate goal is to identify a broader range of predictive markers that can not only guide therapeutic decisions but also help overcome the limitations of current therapies, offering new hope for patients with this challenging malignancy.

Some open questions are still considered by experts on EC, mainly for those with advanced or recurrent disease. Therefore, some critical points concerning unresolved biomarker limitations as well as research gaps need to be answered moving forward. Although the data are strongest for using immunotherapy as part of first-line treatment for people with dMMR tumors, an unsolved question is whether chemotherapy is always needed as part of initial treatment or how long the duration of maintenance treatment should be, since some patients with dMMR tumors do not respond at all to the combined therapeutic regimen. In this way, future research focused on validating emerging biomarkers, coupled with well-designed clinical trials, will be crucial in paving the way for more effective and personalized immunotherapeutic approaches in EC.

## Figures and Tables

**Table 1:** Molecular Subtypes of Endometrial Carcinoma and Their Characteristics.

Feature	*POLE*-Ultramutated (*POLE*-mut)	Mismatch Repair Deficient (dMMR)/Microsatellite Instability-High (MSI-H)	Copy-Number Low (NSMP/No Specific Molecular Profile)	Copy-Number High (CNH/p53-Mutated)
Approx. Frequency	5–8% overall; ~12% in high-grade [21].	~30% overall [22].	~50% overall [4].	~15% overall [4].
Key Molecular Basis	Pathogenic mutations in the *POLE* exonuclease domain.	Defective DNA mismatch repair (somatic or Germline–Lynch Syndrome)	Low frequency of copy number alterations; often PTEN/PIK3CA mutations.	High frequency of copy number alterations; often TP53, PPP2R1A, FBXW7 mutations [12].
Mutational Burden	Ultra-high (Ultramutation) [23].	High (Hypermutation); MSI-H [22,24].	Low [4].	Low [12].
Immunogenicity	High due to numerous neoantigens [23].	High due to numerous neoantigens; high TILs [22,25].	Lower immunogenic potential [4].	Less immunogenic [12].
Predicted ICI Response	Favorable response expected [23,26].	Generally considered the best candidates for ICI therapy [22].	Less likely to respond; ER-negative may respond better (needs validation) [4,27,28].	Less likely to respond to monotherapy; combination strategies explored [12].
Common Associations	Often high-grade tumors, undifferentiated, carcinosarcomas [21].	Endometrioid histology, high TILs, LS association (2–5%) [22].	Typically ER-positive, endometrioid histology, relatively stable genome [4,28].	Often serous or serous-like histology, aggressive clinical course [12,29].
Prognosis	Generally favorable [26].	Variable; dMMR itself can be prognostic [30].	Intermediate [4].	Poorest, associated with 50–70% of mortality [29].

ICI—Immune Checkpoint Inhibitor; TILs—Tumor-Infiltrating Lymphocytes; LS—Lynch Syndrome; ER—Estrogen Receptor.
